# Novel Antimicrobial Treatment Strategy Based on Drug Delivery Systems for Acute Otitis Media

**DOI:** 10.3389/fphar.2021.640514

**Published:** 2021-08-04

**Authors:** Masamitsu Kono, Nafisa K. Umar, Saori Takeda, Makiko Ohtani, Daichi Murakami, Hideki Sakatani, Fumie Kaneko, Denisa Nanushaj, Muneki Hotomi

**Affiliations:** ^1^ Department of Otorhinolaryngology-Head and Neck Surgery, Wakayama Medical University, Wakayama, Japan; ^2^ Department of Otorhinolaryngology, Tokyo Women’s Medical University Medical Center East, Tokyo, Japan

**Keywords:** acute otitis media, drug delivery system, PK/PD, sustained release, antibiotics

## Abstract

Despite tremendous success of pneumococcal conjugated vaccine and antimicrobial treatment by amoxicillin, acute otitis media (AOM) still remains as a great medical concern. Failure of antimicrobial treatment includes several factors. The middle ear cavity is surrounded by bone tissue, which makes it difficult to maintain sufficient concentration of antibiotics. Tympanic membrane of AOM patients thickens and actually becomes a barrier for topical therapy. This review discusses novel antimicrobial treatment strategies based on drug delivery systems (DDS) for AOM. To deliver drugs enough to kill the pathogenic bacteria without systemic side effects, the development of new antimicrobial treatment strategy applying innovative drug DDS has been expected. The sustained-release DDS can achieve sufficient time for antimicrobial concentrations to exceed minimum inhibitory concentration (MIC) for time-dependent antibiotics as well as enough maximum concentration for dose-dependent antibiotics to eradicate causative pathogens in the middle ear. The development of trans-tympanic membranes of DDS, such as hydrogels with chemical permeation enhancers (CPEs), is another attractive strategy. Phage is a promising strategy for developing DDS-based therapies. The DDS formulations enable antimicrobial treatment of AOM by a single dose and thus, an attractive future antimicrobial treatment for AOM.

## Introduction

Acute otitis media (AOM) is one of the leading reasons for prescriptions of antibiotics during childhood (Chonmaitree et al., 2016; Schilder et al., 2016). If not treated adequately, AOM can progress to be recurrent and ultimately result in perforated tympanic membrane or hearing loss (Verhoeff et al., 2005; Bluestone, 1998).

Antimicrobial treatment failure includes anatomical and pathological factors (Costelloe et al., 2010; Venekamp et al., 2015). Antibiotic resistance is one of the greatest challenges in medical care. However, the discovery of new antibiotics stagnates, which brings the urgent demand to develop novel alternative procedures. To deliver drugs enough to kill pathogens without systemic side effects, the development of new antimicrobial treatment strategy applying innovative drug delivery systems (DDS) has been expected. The promising solution for antimicrobial treatment of AOM can be sustained-release or *trans*-tympanic drug delivery.

In this review, we focused on the pathogenesis of antimicrobial treatment failures of AOM and the development of novel antimicrobial treatments based on DDS.

### Principles of Antimicrobial Treatment for the Development of DDS

**FIGURE 1:**
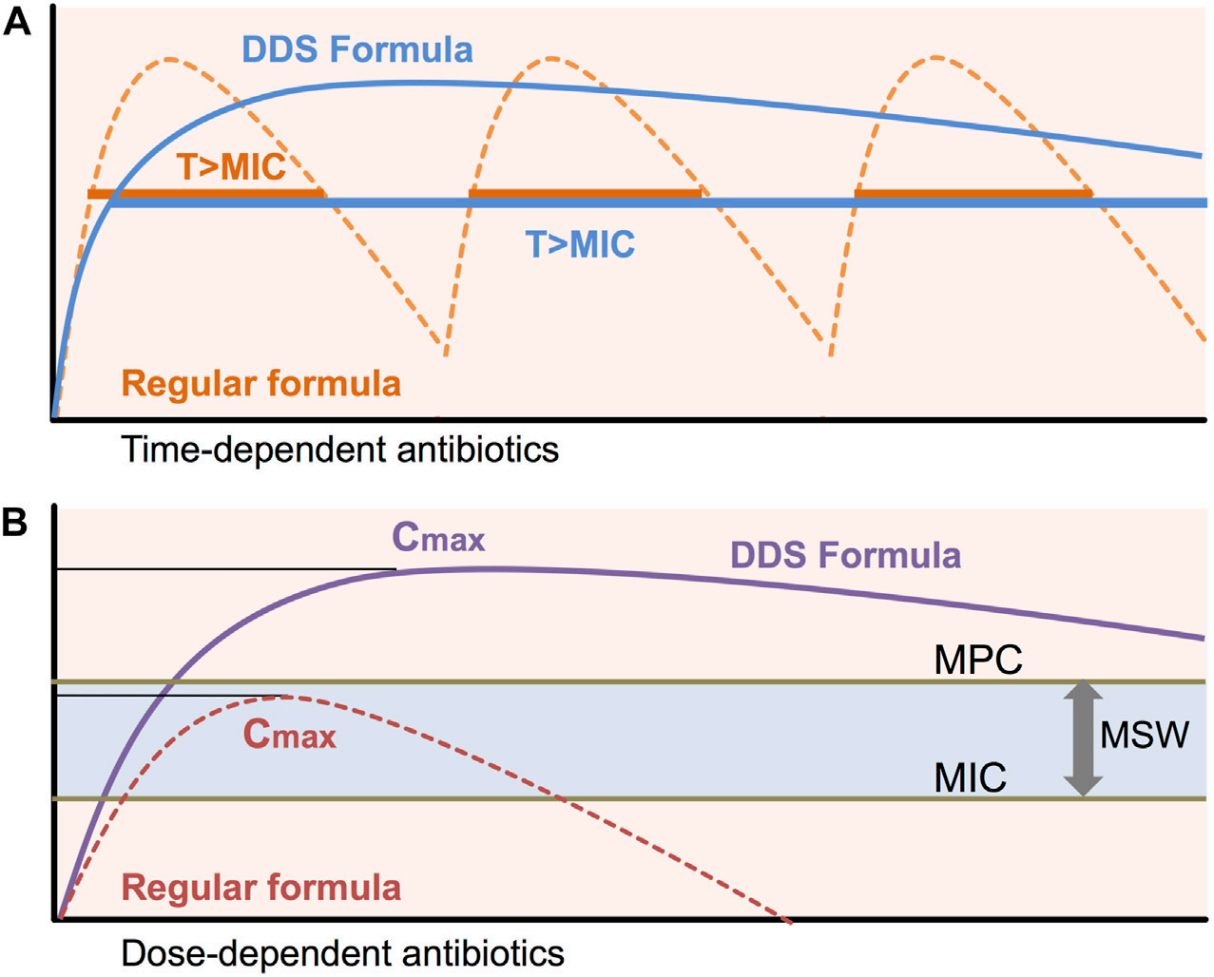
Pharmacokinetics/Pharmacodynamics for DDS To maintain efficient bactericidal activity, the usual preparations of many short half-life antibiotics need to be given frequently. Sub-MIC/MPC concentrations of antibiotics during antibiotic treatment induce antimicrobial resistance. Sustained-release formulations of DDS can maintain constant plasma drug concentrations above MIC (T > MIC) and peak antibiotic concentrations (C_max_). MIC: minimal inhibitory concentration, MPC: Mutation prevention concentration, MSW: Mutant selective window, T > MIC: time above MIC, C_max_: maximum concentration of antibiotics.

To maximize the therapeutic effect and minimize antibiotic resistance, a constant plasma drug concentration over MIC has to be maintained by extended-release formulas. Antimicrobial agents are commonly divided as time-dependent antibiotics and concentration-dependent antibiotics (Craig and Andes, 1996; Dagan, 2007). Time-dependent antibiotics such as β-lactams exert a bactericidal effect when the drug is maintained above the MIC (Jacobs, 2001; Drusano, 1988; Turnidge, 1998). To obtain the advantages of time-dependent antimicrobial agents, sustained-release formulas are ideal alternatives (Figure 1A). Unlike time-dependent antibiotics, concentration-dependent antibiotics such as quinolones show a bactericidal effect depending on the concentration and concentration-dependent post-antibiotics effect (PAE) (Thomas et al., 1998). Sustained-release formulations of concentration-dependent antibiotics can maximize C_max_ and minimize toxicity by releasing the drug in controlled manner (Figure 1B).

### Current Treatment Modalities for AOM

Amoxicillin (AMPC) is recommended as a first-line drug in the antibacterial treatment of AOM (Hoberman et al., 2011; Tähtinen et al., 2011; Venekamp et al., 2015; Harrison, 2017; Hotomi et al., 2021). National guidelines recommend systematic treatment for severe (moderate to severe otalgia and/or fever ≥39°C) or complicated cases regardless of patient age and disease laterality (Lieberthal et al., 2013; Siddiq and Grainger, 2015). The Japanese practical guideline also recommends antimicrobial treatments with AMPC based on the severity of the disease evaluated by a scoring system (Hayashi et al., 2020). However, numerous barriers will restrict efficacy of systemic antimicrobial treatment. The middle ear is encased in a bony cavity, despite rich vascular supply, it is hard to maintain sufficient concentration of antibiotics when given orally (Barry et al., 1997). The concentration of β-lactam antibiotics transferred to the middle ear cavity is at about only 10–15% of the blood concentration (Figueira et al., 2016; Seppanen et al., 2019). In addition, the tympanic membrane of AOM patients becomes thicker, which is a barrier for topical antimicrobial treatment. Biofilm formed by pathogens also reduces the efficacy of antibiotic therapy (Hall-Stoodley et al., 2006; Coates et al., 2008). Strategies to deal with relapsing or recurrent AOM are necessary to be developed.

### Microbiological Factors Associated With Treatment Failures of AOM

Several factors are involved in the antimicrobial treatment failure of AOM (Hotomi et al., 2004; Kono et al., 2020). Otopathogens may persist in middle ear fluids despite intensive antibiotic treatments.

**FIGURE 2:**
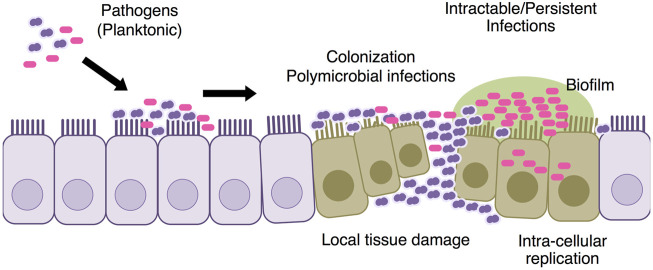
Pathogenesis of intractable AOM The causative pathogen colonizes the mucosal surface of the middle ear. *S. pneumoniae* causes early episodes of AOM and mucosal damage in the middle ear, making children more vulnerable to refractory episodes of latter AOM. Both bacterial biofilm and intra-cellular replication are involved into the intractable AOM.

**Antimicrobial resistance**: Introductions of PCVs have led the change of the etiology of causative bacteria and their antimicrobial resistances; increases of non-vaccine types of pneumococci and NTHi and parallel decreases of pneumococcal AOM (Kaur et al., 2017; Ubukata et al., 2018; Ubukata et al., 2019; Kaur et al., 2021). *Streptococcus pyogenes*, at least in some geographic areas, also play a relevant etiologic role and cause some of the most severe AOM cases. We are concerned by the recent increase in the isolation rate of ß-lactamase non-producing ampicillin resistant *H. influenzae* (BLNAR) (Kakuta et al., 2016). It is true that NTHi is now associated with complex OM, recurrent OM episodes and the development of OM cases (Dagan et al., 2016; Lo et al., 2019). Antimicrobial resistance of *S. pneumoniae* and NTHi can be solved simply by increasing antibiotic dosage or including ß-lactamase inhibitor such as clavulanic acid. However, NTHi being more frequent as a component of multi-bacterial infections than as a single pathogen makes antimicrobial treatments more complex (Grevers et al., 2012; Pichichero, 2016). NTHi remains the most frequently isolated pathogen in children with treatment failure cases of AOM or recurrent AOM (Pichichero et al., 2008). The recurrent, persistent and complex AOM showed severe damages of the middle ear mucosa rather than otitis media with effusion and simple recurrence of AOM (Figure 2).

**FIGURE 3:**
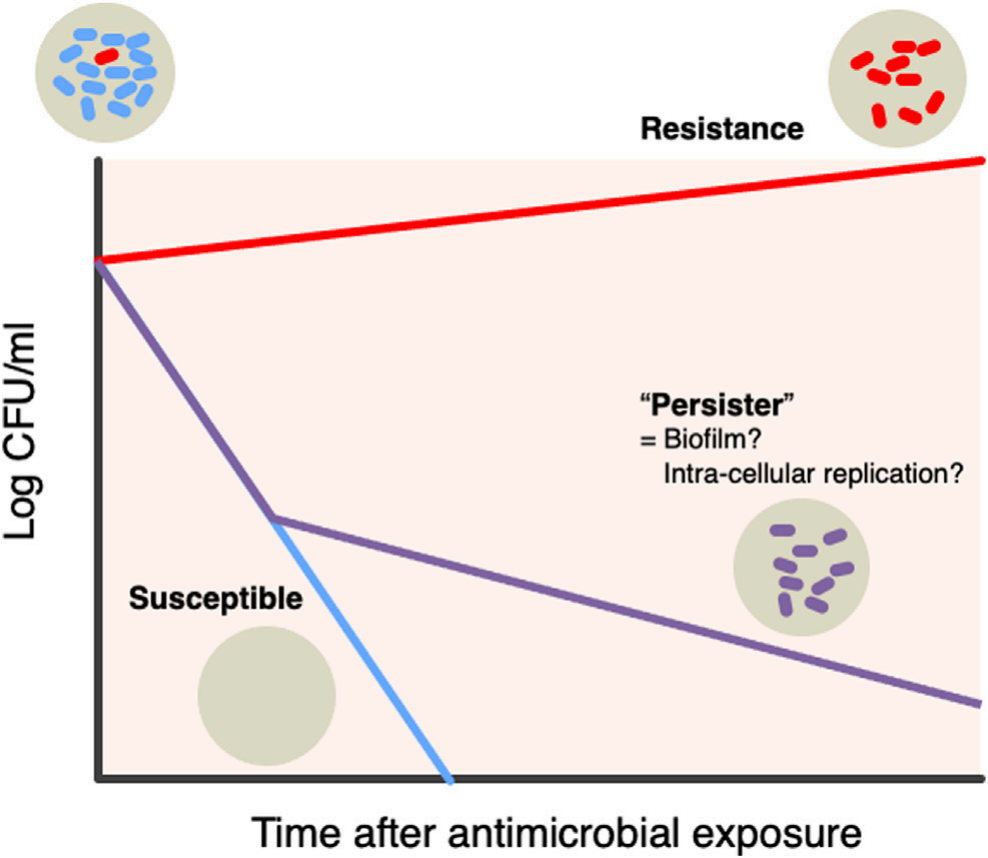
Hypothesis bacterial “persister” There is a specific bacterial subpopulation named “Persister” that can withstand intensive antimicrobial treatments. Persistence is the ability of a subpopulation to survive exposure to bactericidal concentrations of antibiotics, as opposed to the resistance being the ability of bacteria to replicate in the presence of antibiotics. The presence of persistent antibacterial strains is responsible for the failure of bacterial clearance and its impact on the success of antibacterial treatment. Two mechanisms, biofilm and intracellular replication, are involved in the persistent etiology of the infectious diseases.

**Bacterial persistence**: Recent unique hypothesis suggested that there is a certain bacterial subpopulation that can persistently survive intensive antimicrobial treatments (Fisher et al., 2017; Balaban et al., 2019) (Figure 3). Contrast to resistance (the ability of bacteria to replicate under the presence of antibiotics), persistence is the ability of a subpopulation to survive exposure to a bactericidal concentration of antibiotics (Cohen et al., 2013; Levin-Reisman et al., 2017). The presence of antimicrobial persistent strains handles failures of bacterial clearance and influence on the success of antimicrobial treatment. The mechanism of bacterial biofilm formations and intra-cellular residence of bacteria, involves in the persistent pathogenesis of infections (Costerton et al., 1999; Keren et al., 2004; Ercoli et al., 2018).

**Bacterial biofilm**: A problem that hinders efficient drug delivery to treat AOM is biofilm formation (Römling and Balsalobre, 2012; Cuevas et al., 2017; Das et al., 2017). We can associate recurrent cases with biofilm that makes eradication of pathogens significantly more difficult mainly because antibiotics and components of immunity cannot penetrate biofilms and reach pathogens in adequate concentrations. Biofilms lead to evading the host immune system as well as antibiotic resistance (Coticchia et al., 2013). NTHi in biofilm can survive antimicrobial exposures and cause persistent clinical courses of AOM (West-Barnette et al., 2006; Coticchia et al., 2013). Around 84.3% NTHi clinical isolates are biofilm forming strains and those isolated from AOM cases not improved by AMPC produces quite higher biofilm than those improved by AMPC (Moriyama et al., 2009). Co-infection with NTHi and *S. pneumoniae* resulted in higher biofilm formation than single infection by NTHi (Tikhomirovaand Kidd, 2013). The NTHi persists in a biofilm despite intensive antimicrobial treatments, and therefore, even antibiotics with clinically attainable concentration may not adequately clear infections. Because the bacteria surrounded by biofilms are very difficult to remove by AMPC treatment, myringoplasty or tympanostomy tube replacement is considered to remove the glue middle ear effusion containing biofilm and maintain aeration of the middle ear cavity. We also have reported the usefulness of macrolides as a reducer of biofilm among children with AOM (Kono et al., 2021).

**Intra-cellular replication**: Intra-cellular replication of NTHi also plays an essential role in the persistent and recurrent clinical course of AOM of this pathogen (Hotomi et al., 2010). Intracellular localization of NTHi may serve as a reservoir of this pathogen to promote recurrent infections (St Geme and Falkow, 1990; Johnson et al., 1996; Ketterer et al., 1999; van Schilfgaarde et al., 1999; Ahrén et al., 2001). The accumulation of antibiotics is important in the treatment of infections caused by intracellular pathogens. Most antibacterial reagents have been shown to be bactericidal against NTHi *in vitro*. However, ß-lactams neither accumulate in the cytoplasm nor show bactericidal to NTHi internalized into epithelial cells. Ahrén et al. found exhibited high intracellular activity of antibiotics against NTHi with low-dose quinolones but only a limited effect of high-dose ampicillin (Ahrén et al., 2001).

*S. pneumoniae* causes early episodes of AOM and is associated with clinical severity of the infectious disease (Palmu et al., 2004; Barkai et al., 2009). Pneumococci cause mucosal damage of the middle ear and predisposes children to later intractable episodes of AOM (Rodriguez and Schwartz, 1999; Hotomi et al., 2016; Leibovitz et al., 2003). Early infection with *S. pneumoniae* strains facilitates subsequent infections by biofilm formation and intracellular replication (Fisher et al., 2017; Ercoli et al., 2018). The innovative DDSs to eradicate middle ear pathogens need to be developed to control intractable AOM (Dagan et al., 2016).

### Topical Antimicrobial Treatment of AOM

Topical application of antibiotics by eardrops can deliver antibiotics at high concentration directly to the middle ear. However, the antimicrobial treatments can be indicated in particular cases with tympanic membrane perforations or tympanostomy tubes. Current clinical practice guidelines for the treatment of AOM recommend the topical application of antibiotics after tympanostomy tube placement.

A prospective study of AOM patients with otorrhea by ventilation tube revealed that topical use of 0.3% ciprofloxacin ear drop mixed with 0.1% dexamethasone twice daily for 7 days provided a significantly earlier resolution of otorrhea and cure of AOM as compared to AMPC/CVA (600 mg/42.9 mg) per os every 12 h for 10 days (Dohar et al., 2006). Van Dongen et al. reported superior reduction of ear discharge by 2 weeks of topical ear drop treatment with mixture of hydrocortisone, bacitracin, and colistin compared to oral administration of AMPC/CVA (30 mg/7.5 mg/kg/day) for 7 days (rate of continuous ear discharge: 5% topical vs 44% oral) (Van Dongen et al., 2014). In randomized clinical trials (RCTs) comparing topical treatment with antibiotics alone, antibiotics with corticosteroid, and fluoroquinolones with corticosteroids shortened the duration of otorrhea in pediatric AOM patients with tympanostomy tubes (Roland et al., 2004; Schmelzle et al., 2008). As topical treatment can deliver drugs at the site of infection and inflammation, they demonstrate both attainments of local concentrations over 1,000 times higher than those of oral administrations and reduced side effects. However, it is important to consider the potential of topical antimicrobial treatment if tympanic membrane perforation exists.

### Antimicrobial Treatment Strategy Based on Sustained-Release Drug Delivery Systems for AOM

**FIGURE 4:**
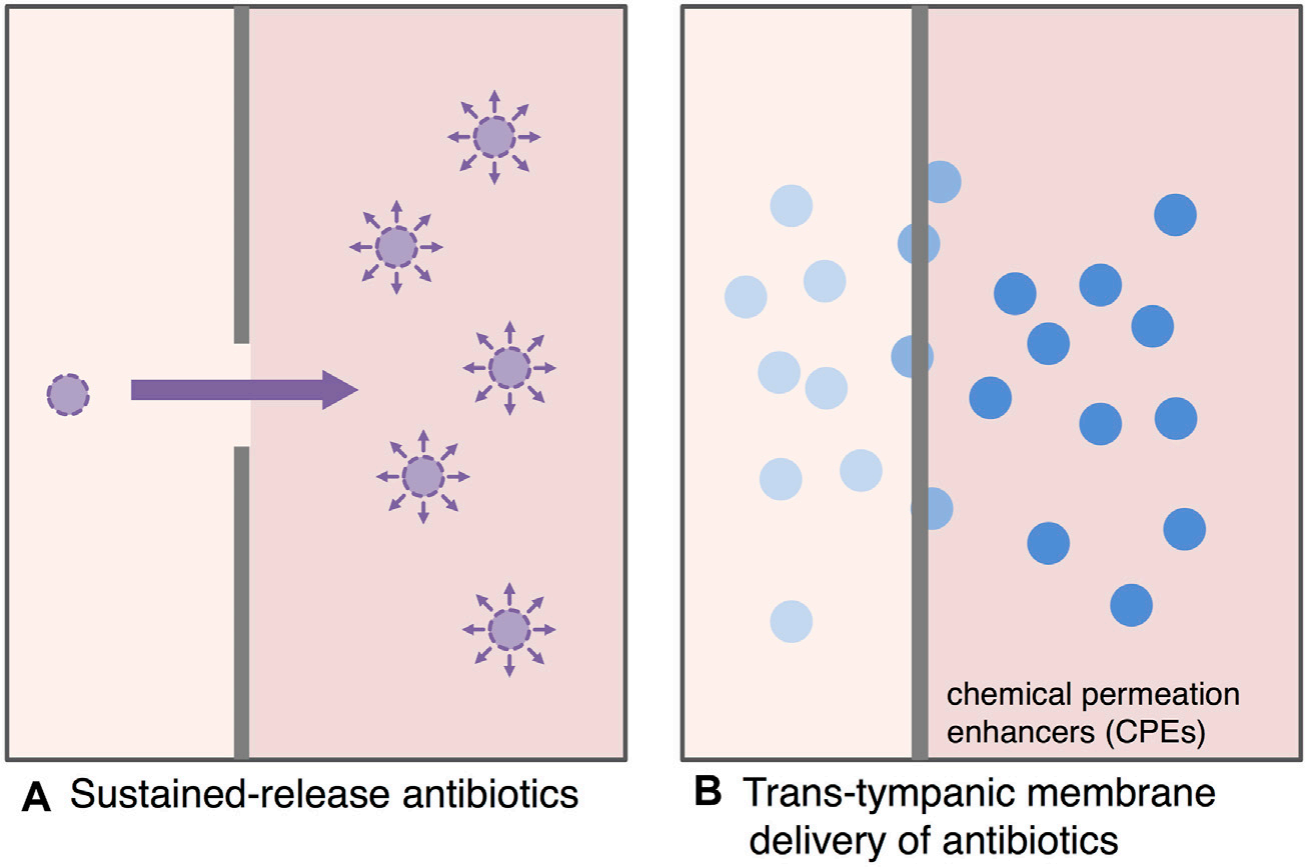
Antimicrobial treatment strategy for AOM based on drug delivery systems. The development of sustained-release antibiotic delivery system and *trans*-tympanic membrane drug delivery systems is a fascinating challenge to maximize the bactericidal effect of antibiotics and to reduce the induction of antibiotic resistance. **(A)**: Sustained-release antibiotics, **(B)**: *Trans*-tympanic membrane delivery of antibiotics.

Development of a sustained-release antibiotic delivery system is one of the fascinating challenges to maximize the bactericidal effect of antibiotics and to decrease the induction of antibiotic resistance (Gao et al., 2011) (Figure 4A). Advanced progress in sustained-release antimicrobial researches was done over the past few years.

Continuous local action of antibiotics directly on the middle ear (the therapeutic target) minimizes the side effects and the concentration limitation of systemic (oral/intravenous) administration significantly. Hence, the extended-release DDS can achieve the sufficient time above MIC as well as enough maximum serum concentration (C_max_) to eradicate causative pathogens in the middle ear.

Two representatives of sustained-release formulation of time-dependent antibiotics have been reported; amoxicillin clavulanate, Augmentin XR, and clarithromycin, biaxin (Gotfried, 2003; Jackson et al., 2006). The sustained-released antibiotics can maintain sufficient time above MIC, significantly extend exposure and bactericidal time during the dosing intervals compared to that enabled by conventional formulations. Clinical trials have shown that the enhanced pharmacokinetics provided sufficient bactericidal efficacy and resulted in higher treatment success rates compared to usual formulations.

As for the dose-dependent antibiotics, sustained-release formulations of ciprofloxacin, Cipro XR or Cipro XL, were developed. The sustained-release formulations of ciprofloxacin can provide a higher C_max_ and performance, at once-daily treatment than the regular twice-daily formulation (Talan et al., 2004).

Recent important progress that will be able to apply for the treatment of AOM is the development of sustained-release materials such as matrix systems, hydrophilic matrix systems, biodegradable materials, coating materials, and nanoparticles. In particular, nanotechnology is being extensively applied to develop drug delivery systems as reviewed by Zhang et al. (2010). Antibiotic-contained nanoparticles can enter host cells via endocytosis and subsequently release contained antibiotics to kill intracellular bacteria that are related with intractable pathogenesis of AOM. In addition, a sustained-release drug delivery system can show efficacy against AOM with tympanic membrane perforation especially when it is recurrent.

### Development of Trans-Tympanic Membrane of Drug Delivery Systems for the Future Antimicrobial Treatment for AOM

Antimicrobial treatment with a drug delivery system through tympanic membrane is one of the new attractive treatment strategies for AOM (Figure 4B). Tympanic membrane, which is only about 100 µm thick, provides a strong impermeable barrier to antibiotics. The development of interesting antimicrobial treatments for AOM is enhancing the permeability of antimicrobials through the tympanic membrane (Khoo et al., 2013). Yang et al. applied hydrogels with chemical permeation enhancers (CPEs) for promising candidate of *trans*-tympanic DDS to overcome the impermeable barrier of tympanic membrane (Yang et al., 2016; Yang et al., 2018a; Yang et al., 2018b). Polyxamers (tri-block copolymers of polypropylene and polyethylene) was evaluated as an attractive candidate for hydrogel DDS, and approved by United States Food and Drug Administration (FDA) (Moghimi and Hunter, 2000; Kabanov et al., 2002). A ciprofloxacin thermosensitive suspension polyxamer 407 gel (Otiprio) for pediatric otitis media with effusion is the first single-dose treatment approved by FDA for post-tympanostomy tube related otorrhea (Edmunds, 2017). In an experimental AOM model of chinchilla, the administration of hydrogel containing ciprofloxacin through external auditory canal effectively eradicated NTHi from the middle ear (Yang et al., 2016).

The hydrogel *trans*-tympanic membrane DDS reduces systemic side effects and suppresses the induction of antimicrobial resistant pathogens by directly targeting the infected sites of the middle ear (without systemic distribution of the antibiotics). To develop the innovative antimicrobial treatment by applying an ideal hydrogel-based DDS, it is important to release the drug maximally and efficiently, have a high gelation efficacy, and obtain a continuous release of antibiotics (appropriately for 7 days). The DDS formulations enable antimicrobial treatment of AOM by a single dose and thus, an attractive future antimicrobial treatment for AOM.

Liposome is also an attractive procedure to develop *trans*-tympanic treatment. Silva et al. reported successful development of a sustained release therapy for pneumococcal endolysin using liposomes composed of L-α-lecithin and sodium cholic acid or PEG2000PE (Silva et al., 2021). The liposome showed better biocompatibility and antibacterial activity against both planktonic and biofilm-forming pneumococci. Continuous innovation in *trans*-tympanic drug delivery including the development of an attractive *in situ* human temporal bone model that characterizes drug permeability of the tympanic membrane is important to develop the effective *trans*-tympanic membrane DDS (Early et al., 2021).

### Future Application of Phage Therapy and Oligonucleotides Therapy

The FDA approved a number of bacteriophage-related products. The first clinical trial of bacteriophage (Biophage-PA) demonstrated safety and efficacy of a phage therapy against *P. aeruginosa* (Wright et al., 2009). However, they are not applied to treatments for AOM until now. The bacterial phage therapy can be applied to either *trans*-tympanic membrane or direct injection by tympanocentesis or myringotomy. The ability of a bacteriophage to pass through a tympanic membrane is evaluated by amino acid sequences of its surface structure (Wright et al., 2009). Kurabi et al. showed 22 unique phage clones could penetrate the tympanic membrane based on the phage determination (Kurabi et al., 2016). Recent efforts to improve drug delivery to the middle ear have focused on optimizing peptide sequences that are actively transported through the tympanic membrane (Kurabi et al., 2018).

Oligonucleotides also provide antibacterial activity by knocking down the expression of essential genes of pathogens required for survival or virulence (Streicher, 2021). There are two approaches such as antisense oligonucleotides and short interfering RNAs. The antimicrobial oligonucleotides can knock down essential genes. Delivery of these oligonucleotides presents the greatest challenge to their therapeutic success.

### Conclusion and Future Directions

Topical antimicrobial treatments, whether combined with systemic antimicrobial treatments or not, have the potential to achieve higher efficiency in antimicrobial delivery. With current successful delivery of antimicrobial agents through the tympanic membrane, we can expect to apply not only to antibiotics but also for other antimicrobials such as peptides, antibodies and phages etc. Further studies are needed to elucidate the mechanism of *trans*-tympanic membrane transport. There is also a need to understand the etiology of AOM, especially recurrent AOM. These provide new clues for the development of effective preventive and therapeutic strategies. For effective treatment of AOM, non-surgical drug delivery to the middle ear presents challenges that need to be considered. The novel field of phage therapy will help in the development of new and effective treatments for AOM.
